# Preparation of the Hybrids of Hydrotalcites and Chitosan by Urea Method and Their Antimicrobial Activities

**DOI:** 10.3390/polym11101588

**Published:** 2019-09-28

**Authors:** Bi Foua Claude Alain Gohi, Hong-Yan Zeng, Xiao-Ju Cao, Kai-Min Zou, Wenlin Shuai, Yi Diao

**Affiliations:** 1 Biotechnology Institute, College of Chemical Engineering, Xiangtan University, Xiangtan 411105, China; gohibifouaca@smail.xtu.edu.cn (B.F.C.A.G.);; 2 School of Biological and Chemical Engineering, Panzhihua University, Panzhihua 617000, China; diaoy163@163.com; 3 College of Chemistry and Chemical Engineering, Xinjiang University, Urumqi 830046, China; swlswlswl123456789@163.com

**Keywords:** chitosan, ZnAl hydroxide, hybrid, urea method, chitosan amount, antimicrobial activity

## Abstract

Hybrid nano-supra molecular structured materials can boost the functionality of nano- or supra-molecular materials by providing increased reactivity and conductivity, or by simply improving their mechanical stability. Herein, the studies in materials science exploring hybrid systems are investigated from the perspective of two important related applications: healthcare and food safety. Interfacing phase strategy was applied, and ZnAl layered double hydroxide-chitosan hybrids, prepared by the urea method (U-LDH/CS), were successfully synthesized under the conditions of different chitosan(CS) concentrations with a Zn/Al molar ratio of 5.0. The structure and surface properties of the U-LDH/CS hybrids were characterized by X-ray diffraction (XRD), Fourier-transform infrared spectrometer(FTIR), scanning electronmicroscopy (SEM), ultravioletvisible (UV-Vis), and zero point charge (ZPC) techniques, where the effect of CS concentration on the structure and surface properties was investigated. The use of the U-LDH/CS hybrids as antimicrobial agents against *Escherichia coli*, *Staphylococcus aureus*, and *Penicillium cyclopium* was investigated in order to clarify the relationship between microstructure and antimicrobial ability. The hybrid prepared in a CS concentration of 1.0 g∙L^−1^ (U-LDH/CS_1_) exhibited the best antimicrobial activity and exhibited average inhibition zones of 24.2, 30.4, and 22.3mm against *Escherichia coli*, *Staphylococcus aureus*, and *Penicillium cyclopium*, respectively. The results showed that the appropriate addition of CS molecules could increase antimicrobial ability against microorganisms.

## 1. Introduction

The use of nanostructures is known to achieve levels of functionality not possible to reach when using bulk materials [1]. It is in this context that the notion of hybrids has been introduced, which is a strategy that aims to combine different structures in order to obtain a more efficient one. An infinite number of possibilities can emerge from the combination of phases in hybrid systems [2]. Different types of hybrid materials have been studied, including organic hybrids, inorganic hybrids, and organic-inorganic hydrids. Organic hybrid systems still dominate several traditional areas of chemical science and well-known applications, such as the synthesis of pharmaceutical compounds and drugs [3]. However, the mechanical properties (resistance to temperature and environmental stability) of the latter limits their application fields [4]. The applications of hybrid inorganic materials range from ion exchangers, semiconductors, adsorbent, electrochemical sensor catalysts, and catalyst support [5,6]. The toxicity and low biocompatibility of organic-inorganic hybrids also limit their application in biomedical science and drugs production. Among the techniques used for the preparation of hybrids, mention may be made, inter alia, of: Directintercalation, in-situ polymerization, intact melt-blending, and surface-modified blending. Organic-inorganic hybrids overcome the limitations of the previous two by their non-toxicity, their biocompatibility, and the strengthening of their mechanical properties [7,8]. Bacterial pathogens are one of the primary causes of human morbidity worldwide [9]. Historically, antibiotics have been highly effective against most bacterial pathogens; however, the increasing resistance of bacteria to a broad spectrum of commonly used antibiotics has become a global health-care problem. In recent years, the use of hybrid materials with a wide range of properties and applications has increased considerably. Construction of organic-inorganic hybrid materials is a rapidly expanding field in materials chemistry for the design of advanced materials with specific structure and functionality [10]. Bio-inorganic hybrids may exhibit, not only a combination of properties from the disparate components, but also further enhanced property tunability and new synergistic properties that arise from the interactions between the biological molecules and inorganic materials [11]. Among various hybrids of inorganic and organic materials, layered double hydrotalcites (LDH) and chitosan (CS) are of particular interest due to their wide applications range. LDH and CS have received considerable attention recently due to their wide applications in a variety of areas, including chemistry, physics, materials science, and the biomedical science [12,13,14]. Chitosan (CS) is a naturally occurring, cationic polysaccharide composed of (1,4)-linked 2-amino-2-deoxy-β-D-glucose and 2-acetamido-2-deoxy-β-Dglucose units. Chitin is the transformed base material resulting in chitosan, which is obtained from shrimp, crab, and lower plants and animals. By changing different parameters, one can achieve the desirable targeted chitosan molecule without changing the chemical compositions [15].CS has three types of functional nucleophile groups consisting of a C-2 NH_2_ group, a secondary C-3 OH group, and a C-6 primary OH group. CS is a biopolymer that presents reactive functional groups that are susceptible to chemical modification, and has been shown to be a functional polymer that can covalently graft antioxidant/antimicrobial activity onto its backbone [16].CS has shown interesting antibacterial and antifungal activities against a wide range of microorganisms when compared to other polymers and biopolymers [17].Layered double hydroxides (LDH) are composed of positively charged brucite-like layers of divalent and trivalent metal hydroxides whose excess positive charge is compensated by anions and water molecules present in the interstitial position. Among the heterostructured nanomaterials, layered nanohybrids have received intense attentions in many areas due to their unique physico-chemical and mechanical properties that cannot be obtained from other analogous nanohybrids [18]. Moreover, LDH has essential properties, such as biocompatibility, null toxicity, and allergenicity [19]. They can be represented by the general formula:

$$[M{\left(\mathrm{II}\right)}_{1-x}M(\mathrm{III}{)}_{x}(\mathrm{OH})2{]}^{x+}[A_{x/n}^{n-}{\mathrm{YH}}_{2}O{]}^{x-},$$

where M(II) = Mg, Ni, Co, Cu, Zn, Mn; M(III) = Al, Fe, Cr, V; A^n−^ = ${\mathrm{CO}}_{3}^{2-}$, Cl-, ${\mathrm{SO}}_{4}^{2-}$, etc., and x = 0.1–0.35.

ZnAl hydroxides are effective antimicrobial agents for bacteria such as *Escherichia coli* and *Staphylococcusaureus* due to the hydroxides (–OH) and the nature of the metallic cations, where Zn^2+^ is one of the most active ones, due to its strong oligodynamic features [20]. Zinc and CS have excellent antibacterial activity [21,22]. The goal of layered double hydroxide-chitosan hybrids, prepared by the urea method (U-LDH/CS) preparation was to assess the possible antimicrobial activity of pure CS macromolecules under microbial culture medium pH conditions and to explore the impact of U-LDH on CS through the inorganic-organic hybrid. Since the antimicrobial properties of CS are limited to pH values below six [23,24,25,26], hybrid materials that have the ability to kill pathogenic bacteria and prevent bacterial colonization are desired for utilization in several application areas such as food-contact materials, food packaging, textiles, water purification systems, prosthetic devices, and hospital equipment surfaces. The aim here is to activate and reinforce the antimicrobial activity of pure chitosan without taking into account both the conditions of the bacterial culture medium and its molecular weight by integrating it into a hybrid structure. In this study, using interfacing phases, a surface-modified blending process that is a strategy used to obtain a set of properties in one system that are beyond the abilities of single phases [2], the LDH/CS hybrids as antimicrobial agents were prepared in different CS concentrations by urea method, where CS was used as a soft template. In order to clarify the relationship between the microstructure and antimicrobial ability, the physico-chemical properties of the LDH/CS hybrids were characterized by XRD, FTIR, SEM, UV-Vis and pH of zero point charge (pHzpc) measurements. In addition, the antimicrobial activity was also evaluated.

## 2. Materials and Methods

### 2.1. Experimental Materials

Chitosan (CS, DA ≥ 91%, MW 1.5.10^5^ kD) was purchased from Sinopharm Chemical Reagent Co. Urea (CON_2_H_4_), Al(NO_3_).9H_2_O, and Zn(NO_3_)2·6H_2_O, KOH, ethanol were purchase from Heng Xing Chemical preparation Co. Ltd. (Tianjin, China). NaOH was purchase from Xilong Chemical Co., Ltd. (Guangdong, China) and tetracycline powder (TC) was purchased from Sigma–Aldrich (Shanghai, China).

### 2.2. Preparation of ZnAl Hydroxide and CS Hybrids

#### 2.2.1. Preparation of the ZnAl Hydroxide by Urea Method

For the sake of comparison, ZnAl hydroxide (U-LDH_5_) with a Zn/Al molar ratio of 5.0 was prepared using the urea method proposed by Zeng et al. (2009) [27], with slight modifications. Zn(NO_3_)_2_·6H_2_O and Al(NO_3_)_3_·9H_2_O (total amount of metal ions was 0.12 mol) were mixed with a set up Zn/Al molar ratio by using 600 mL deionized water. The mixed solution was poured into a three-necked round bottomed flask, and urea (urea/NO_3_^−^ molar ratio of 4: 1) was added. The reaction solution was magnetically stirred at 110 °C for 12 h. Then the resulting reactant was crystallized statically at 80 °C for another 12 h. The precipitate was centrifuged and washed thoroughly with deionized water and was subsequently dried at 80 °C overnight. The dried material was denoted as LDH. For convenience, the resulting sample with the Zn/Al molar ratio of 5:1 was designated as U-LDH_5_.

#### 2.2.2. Preparation of the U-LDH_5_ and CS Hybrids

The hybrids (U-LDH/CS) of the LDH and chitosan (CS) were prepared under the conditions of different CS concentrations and a Zn/Al molar ratio of 5.0 by the urea method. The U-LDH_5_ and CS hybrids were prepared using CS molecules as a soft template. CS solutions at the CS concentrations of 0.5, 1.0, 1.5, 2.0, and 3.0 g∙L^−1^ were separately obtained by dissolving powered CS in the solution containing 1% (w/v) acetic acid. During the preparation of the hybrid, 400 mL of mixed salt solution containing Zn(NO_3_)_2_∙6H_2_O (0.15 mol∙L^−1^), Al(NO_3_)_3_∙9H_2_O (0.03 mol∙L^−1^), and urea (urea/NO_3_^−^ molar ratio of 4.0) was placed into a three-necked flask. 100 mL of CS solution was dropped into the mixed salt solution under stirring (300 rpm) in pH 8.5 at room temperature. After dripping, the reaction temperature was raised to 103 °C for 12 h under stirring. After the reaction, it was crystallized at 80 °C for 18 h, filtrated, washed, and then dried at 90 °C for 6 h, and was denoted as U-LDH/CS. For convenience, the resulting products prepared in the CS solutions of 0.5, 1.0, 1.5, 2.0, and 3.0 g∙L^−1^ were designated as U-LDH/CS_0.5_, U-LDH/CS_1_, U-LDH/CS_1.5_, U-LDH/CS_2_, and U-LDH/CS_3_, respectively.

### 2.3. Characterization Techniques

It should be noted that 21 samples were analyzed and tested, including hybrid preparation methods and analytical measurements, all in triplicate.

#### 2.3.1. XRD Analyses

X-ray powder diffraction (XRD) analysis was performed on a Rigaku (Tokyo, Japan) D/MAX-2500/PC with Cu Ka radiation (λ = 1.5405 Å), with an operating voltage of 40 kV, a current of 30 mA, a scan rate of 2°min^−1^, and a scanning range from 5 to 90 °2θ, in steps of 0.0334° with a counting time per step of 650 s.

#### 2.3.2. FTIR Analyses

Fourier-transform infrared (FTIR) spectra of the samples were obtained with a Perkin–Elmer Spectrum One B instrument (Shanghai, China). Powder samples were molded in KBr pellets. 1 mg of the powdered samples was carefully mixed with 250 mg of KBr (infrared grade) and pelletized under a pressure of 10 t for 1 min. The pellets were analyzed to collect 32 scans in the range of 4000–400 cm^−1^ at a resolution of 2 cm^−1^.

#### 2.3.3. SEM Analyses

Scanning electron microscopy (SEM) was carried out using aJSM-6700F microscope (JEOL, Tokyo, Japan) operating at15 keV. Prior to analysis, the samples were covered with gold to avoid charge effects. Samples were sputtered using ion sputtering technology at a magnification of 15,000.

#### 2.3.4. UV-Vis Analysis

UV-visible (UV-Vis) spectra were recorded by spectrophotometer (Shimadzu UV-2550, Kyoto, Japan). The range of wavelengths was 200~800 nm using high-purity BaSO_4_ as a reference.

#### 2.3.5. Point Zero Charge Analysis

The determination of the pH point of zero charge (pHzpc) of the materials was carried out using the potentiometric titration (PT) method described by Li et al. [28]. The pH at pHzpc was determined in NaCl solutions (inert electrolytes) with different concentrations. The experiments were carried out in a shaker at 150 rpm and 25 °C for 200 min. After the experiments, the pH in the solution was measured while a 0.1 mol·L^–1^ NaOH solution was added. The adsorption amount of H^+^ (*Γ_H+_*) and OH^−^ (*Γ_OH−_*) was calculated. Finally, PT curves were obtained by plotting (*Γ_OH−_ − Γ_H+_*) versus pH in NaCl solutions with different concentrations, and the crossover point of (*Γ_OH−_ − Γ_H+_*) ~ pHzpc curves was pHzpc, which was electrically neutral. The permanent charge density (*σp*) at pHzpc was as follows: [29].

*σp = F(Γ_OH−_ − Γ_H+_)zpc/S_BET_* (1)

where, S_BET_ and F are the specific surface area of the samples and the Faraday constant (96485 C∙m^−2^), respectively.

#### 2.3.6. Rheological Analysis

Rheological compression measurements were carried out according to the norm established by Yi et al. [30]. Circular disk-like samples with a 25 mm diameter and a 2 mm thickness were prepared on a compression mold. Rheological studies were carried out on a controlled strain rheometer, (MCR 301, Anton Paar, Austria). The dynamic (frequency sweep) tests were performed at a strain of 0.5%. Storage modulus (G′) and loss modulus (G″) were measured in the frequency sweep experiments performed over a frequency range of 0.1–100 rad/s, with data collected at five points per decade.

#### 2.3.7. Thermogravimetric Analysis

Thermogravimetric and differential thermal gravimetric analyses (TGA-DTG) for pure U-LDH and the U-LDH composites with various CS contents was conducted on a Perkin–Elmer TGA 7-thermal analyzer (Arizona, Waltham, MA, USA) under nitrogen at a purge rate of 20 mL/min, with the scanning temperature in the range from 50 to 800 °C. A platinum crucible with a heating rate of 10 and 12 °C /min and a sample weight of 63.3 mg each were used. Meanwhile, the U-LDH content in the CS composite sample was determined on the basis of the residual ash percentage and the water amount of the U-LDH/CS itself, which measured the weight loss between 200 and 400 °C.

### 2.4. In Vitro Antimicrobial Assay

#### 2.4.1. Microorganism and Media

The bacteria *Escherichia coli* (*E. coli*, Gram-negative bacteria, ATCC 35218), *Staphylococcus aureus* (*S. aureus*, Gram-positive bacteria, ST398) and *Penicilinum cyclopium* (*P. cyclopium*, fungi, AS 3.4513) as target organisms were from the Xiangtan University General Microbiological Culture Collection Center. The *P. cyclopium* strain was maintained in potato dextrose (PD) medium (Qingdao Hope Bio-Technology Co., Ltd., Qingdao, China) at 28 °C for 72 h. The *E. coli* and *S. aureus* strains were maintained in mineral salt (MS) media (Boyao Biotechnology Co., Ltd., Shanghai, China) in pH 7.0 at 37 °C for 24 h. PD and MS are the most widely used media for growing fungi and bacteria [31,32]. All the samples were measured to obtain their OD_600nm_ values for calculating the bacteriostatic concentration. The controlled test contained the nutrient medium with bacterial suspension but without antimicrobial agents.

Mineral salt (MS) agar plate medium (g∙L^−1^): (NH4)_2_SO_4_ 2.0, NaCl 5.0, K_2_HPO_4_ 1.0, KH_2_PO_4_ 1.0, MgSO_4_ 0.1, CaCl_2_ 0.1, and agar 20 at initial pH 7.0. and PD agar plate medium (g∙L^−1^): potato 20.4, dextrose 20.4, and agar 20. All media were sterilized by autoclaving at 121 °C for 25 min.

#### 2.4.2. Antimicrobial Test

The cultures of the bacteria and fungi strains were activated twice in liquid potato dextrose and MS media, respectively, and then the culture at exponential growth phase (OD_600_ around 1.2 absorbance units). All antimicrobial experiments using the three cultures were performed in 250 mL sterile shaking flasks (or 90 mm agar plates), where 100 mL cultures of *P. cyclopium*, *E. coli*, and *S. aureus* strains were transferred into the PD and MS media in sequence for the antimicrobial tests.

Antimicrobial tests were performed using an agar diffusion test method [33]. In this assay, 100 mL microbial suspensions were prepared, and50 μL of a bacterial suspension of *E. coli*, *S. aureus*, and *P. cyclopium* was inoculated evenly onto agar plates. The thin tableting of the sample with 1.0cm diameter was placed into an agar plate with a bacterial suspension. After incubation of *E. coli* and *S. aureus* strains at 37 °C for 24h and *P. cyclopium* strain at 28 °C for 72h, the zone of inhibition around the mesh samples was measured with digital vernier calipers in millimeters (mm). The zone of inhibition of the samples was measured in four directions and reported as a mean value and calculated by Equation (1),

A= (D − d)/2(2)

where A is the zone of inhibition, D is the total diameter of the thin tableting with inhibition area after incubation, and d is the diameter of the thin tableting of the sample before incubation. In this assay, the agar plates with different bacterial suspensions were used as reference samples.

## 3. Results and Discussion

### 3.1. Characterization of the U-LDH/CS Hybrids

#### 3.1.1. XRD Analyses

**Figure 1:**
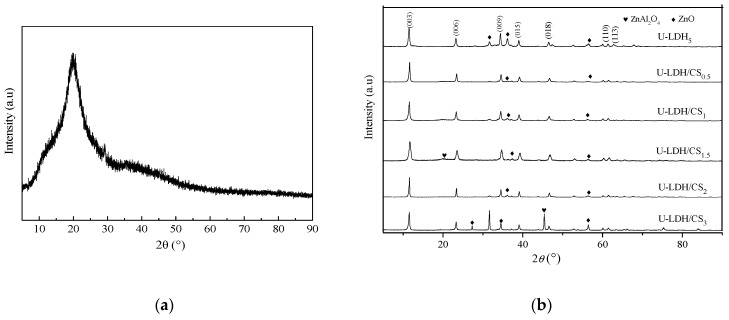
(**a**) XRD pattern of pure CS; (**b**) XRD patterns of the U-LDH_5_ and U-LDH/CS hybrids.

The XRD pattern of pure CS molecules is showed in Figure 1a, where there is a strong reflection in the diffractogram of chitosan at 2θ=21.1, corresponding to the high crystallinity of chitosan. The powder XRD patterns of the U-LDH5, U-LDH/CS_0.5_, U-LDH/CS_1_, U-LDH/CS_1.5_, U-LDH/CS_2_, and U-LDH/CS_3_ hybrids are shown in Figure 1b. There is a typical layered double hydroxide structure with sharp and intense (003), (006), (009), (110), and (113) reflections and broadened (015) and (018) reflections with impure phase ZnO in all six samples, where only the U-LDH/CS_1.5_ and U-LDH/CS_3_ include impure phase ZnAl_2_O_4_. As seen in Table 1, the interlayer distances (d003 about 0.76 nm) for the samples were typical of CO_3_^2−^ pillar hydroxide, indicating that CS molecules were not intercalated into the interlayer spaces between the brucite sheets. All the lattice parameters of the U-LDH/CS samples were the same as those of the U-LDH_5_, revealing that the incorporation of CS did not change the structures of the U-LDH/CS hybrids, which still maintained the native structures. The results demonstrate that the adding of CS as a soft template is conducive to the formation of a heterojunction structure, ZnO-Zn(OH)_2_, which leads to high antimicrobial activity against bacteria combined with high adhesiveness from the CS molecules.

#### 3.1.2. FTIR Analyses

**Figure 2:**
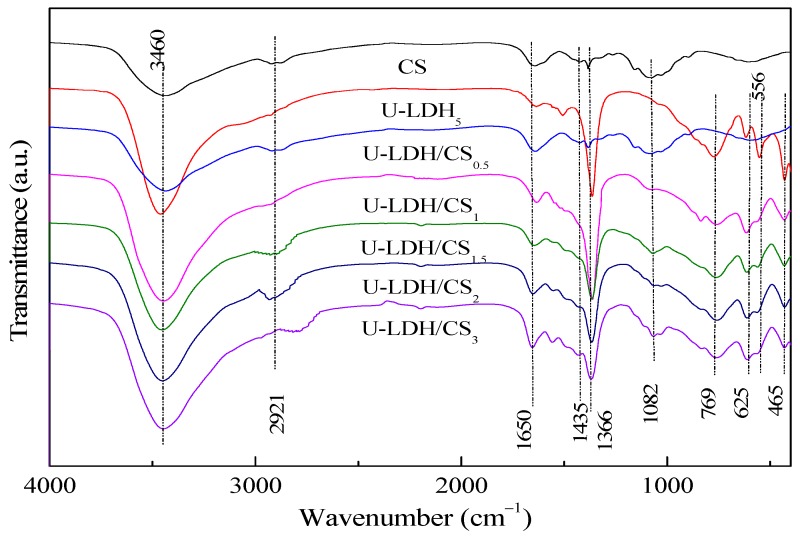
FTIR spectra of pure CS, U-LDH_5_, and U-LDH/CS hybrids.

The FTIR spectra of the pure CS, U-LDH_5_, and U-LDH/CS samples in the range of 4000–400 cm^−1^ are displayed in Figure 2. As can be seen from Figure 2, all samples show significant absorption peaks at about 3460, 1650, and 1366 cm^−1^ [34,35]. For the pure CS, these three peaks belong to the stretching vibration of –C–OH or the stretching vibration absorption peak of –N–H, the absorption peaks of amide I and amide II [36], and carboxylic acid (–C=O) or C–OH [37], respectively. Concerning U-LDH_5_, the peeks are attributed to –OH stretching vibration and interlayer H_2_O absorption for 3460 cm^−1^, bending vibration of adsorbed water and inter-laminar structure water for 1650 cm^−1^, and ν3 stretching vibration characteristic of CO_3_^2−^ at 1366 cm^−1^. In addition to those three common peaks, pure CS at 2921 cm^−1^ has a stretching vibration peak of symmetrical or asymmetric –CH_2_ [38] on a pyranose ring of the chitosan molecule and an oscillation absorption peak of –OH and –CH on the pyranose ring at 1435 cm^−1^ [39], and an absorption peak of –COC– on the glycosidic bond at 1082 cm^−1^. Regarding U-LDH_5_, its FTIR presented a vibrational absorption peak of the lattice layer M–O (Zn–O, Al–O) of the main body of the hydrotalcite in the range of 1000–400cm^−1^. The characteristic absorption peaks of chitosan molecules appear at about 2921, 1435, and 1082 cm^−1^ in U-LDH/CS_0.5_, U-LDH/CS_1_, U-LDH/CS_1.5_, U-LDH/CS_2_, and U-LDH/CS_3_ compared to U-LDH_5_, indicating that chitosan and ZnAl-LDH are organically bound to CS and form hydrotalcite-chitosan composite material.

#### 3.1.3. SEM Analysis

**Figure 3:**
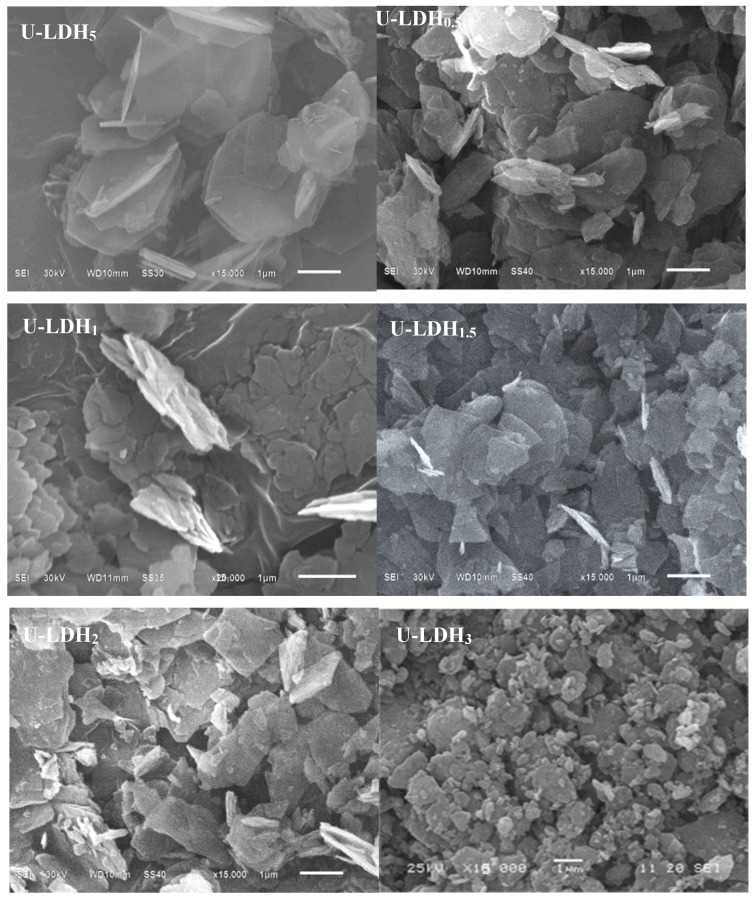
SEM images of the U-LDH_5_ and U-LDH/CS samples, ×15,000.

In order to investigate the morphology of the hybrids, the U-LDH_5_ and U-LDH/CS hybrids were observed by SEM, and the results are shown in Figure 3. As shown in Figure 3, all samples (U-LDH_5_ and U-LDH/CS) exhibit a typical hydrotalcite laminate structure, which is typical of LDH. There is a certain degree of aggregation, which is due to the addition of CS leading to the U-LDH_5_ bonding accumulation. Obviously, CS has an effect on the microstructure of the U-LDH/CS hybrids.

#### 3.1.4. UV-Vis Analyses

**Figure 4:**
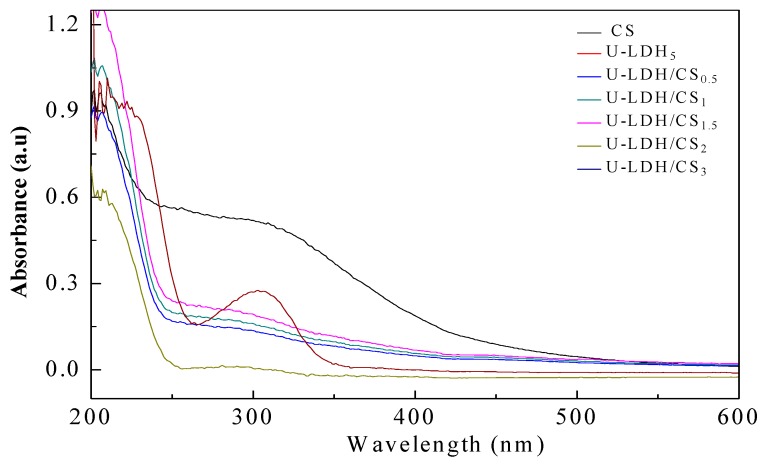
UV-Vis DRS spectra of the pure CS, U-LDH_5_, and U-LDH/CS samples.

UV-Vis measurement is a very simple method that is used to probe the possible changes in the molecule structure of materials. As can be seen from Figure 4, CS exhibits a strong absorption band in a wide range from 230 to 500 nm, with the electronic transition of n → σ * and n → π * belonging to –NH_2_ at ~230 nm and –C=O or –COOH between 220 to 500 nm. U-LDH/CS samples show a weaker absorption band at 250–400 nm, which is due to the complexation of CS with U-LDH, which makes U-LDH/CS exhibit the absorption characteristic of chitosan UV-Vis. On the other hand, with the increase of the amount of chitosan, the absorption intensity of U-LDH/CS in this range also increases. At the same time, the intensity in the absorption band increases, with CS concentration arriving at the highest for U-LDH/CS_1.5_. With a further increase of the CS concentration, the intensity gradually decreases. This suggests that CS has been incorporated into the U-LDH/CS hybrids and impacts the structure of the U-LDH/CS hybrids. This further confirms that CS and ZnAl-LDH have been combined to form the U-LDH/CS complex.

**Figure 5:**
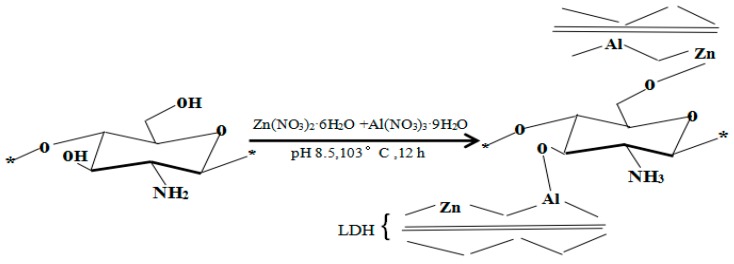
Synthetic scheme of U-LDH/CS hybrids.

Based on the XRD, FTIR, and UV-Vis characterization analyzes, supported by studies on “Antimicrobial Chitosan and Chitosan Derivatives” by Sahariah and Masson [23] and “Polymer-inorganic supramolecular nanohybrids for red, white, green, and blue applications” by Park et al. [40], the probable structure of the hybrid could be presented as the scheme in Figure 5.

#### 3.1.5. Point Zero Charge Analysis

**Figure 6:**
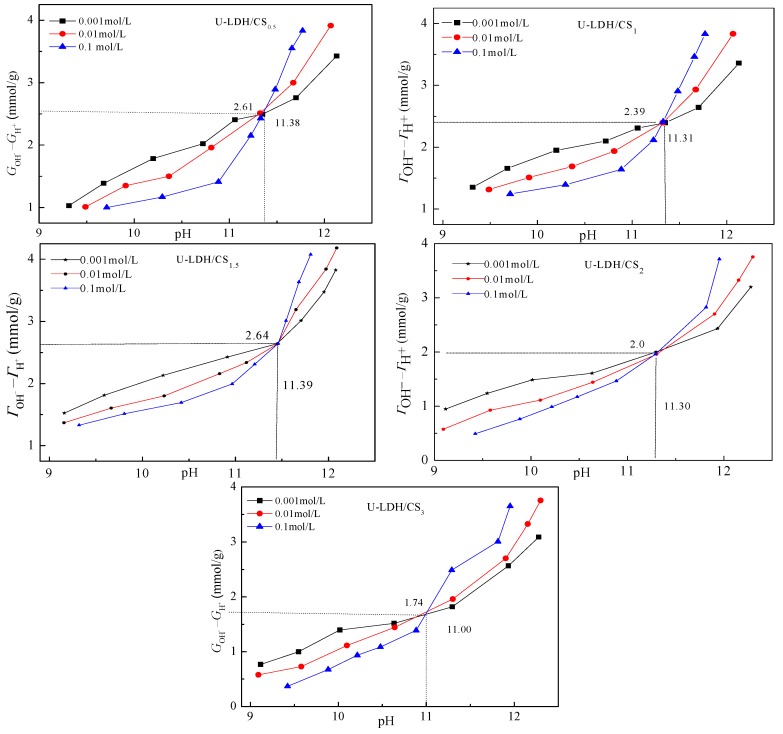
Potentiometric titration curves of the U-LDH/CS samples.

Figure 6 shows the relationship between (*Γ_OH_*−*Γ_H+_*) and the pH of U-LDH/CS. The difference between the adsorbed amount of OH^−^ and H ^+^ on the surface of solid particles (*Γ_OH_*−*Γ_H+_*) is positive, indicating that the sample surface is alkaline with a permanent positive charge. The point zero-charge-pHpzcs of U-LDH/CS_0.5_, U-LDH/CS_1_, U-LDH/CS_1.5_, U-LDH/CS_2_, and U-LDH/CS_3_ were 11.38, 11.31, 11.39, 11.30, and 11.00, respectively. Among them, pHpzcs of U-LDH/CS_1.5_ was the highest. According to Equation (2), the surface permanent positive charge density (σp) of U-LDH/CS_0.5_, U-LDH/CS_1_, U-LDH/CS_1.5_, U-LDH/CS_2_, and U-LDH/CS_3_ were 3.59, 3.47, 3.55, 3.71, and 3.55 C/m^−2^, respectively. The shifts in pHpzc and *σp* of the U-LDH/CS hybrids to higher values were believed to be linked with the incorporation of CS molecules.

#### 3.1.6. Rheological Analysis

**Figure 7:**
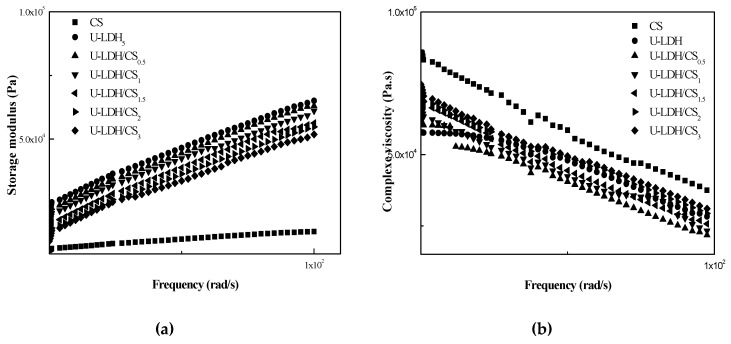
Storage modulus G′ (**a**) andloss modulus G″ (**b**) of the pure CS, U-LDH_5_, and U-LDH/CS samples.

Rheological properties are indicative of melt-processing behavior in unit operations such as injection molding. The linear viscoelastic responses of pure CS and the U-LDH hybrid in melt state were studied with the help of dynamic oscillatory shear measurements, and were reported in terms of storage modulus (G′) (Figure 7a) and complex viscosity (ŋ*) (Figure 7b). At a fixed frequency, the (G′) values of the hybrids are higher than those of pure CS, however, the (ŋ*) values of hybrid are lower than those of pure CS. Consequently, the decrease of the viscosity (ŋ*) has gone up with the increase of U-LDH content. The viscous response of the material is stronger than the elastic response. These results demonstrate that the incorporation of U-LDH could reduce the viscosity properties of the CS matrix. The viscoelastic response in the low and medium frequencies region indicate that the hybrids have an obvious difference in their behavior from that of pure CS. One behavioral difference is that with the increase of CS content, the dependence of (ŋ*) on frequency becomes weaker, while the dependence of (G′) on frequency becomes stronger in the low frequency region. This non-terminal behavior may be attributed to the fact that the intercalated U-LDH layers weaken the mobility of the CS chains and then restrict the long-range relaxation of them [41,42]. U-LDH/CS_3_ shows a fragility to remain compact.

#### 3.1.7. Thermogravimetric Analysis

**Figure 8:**
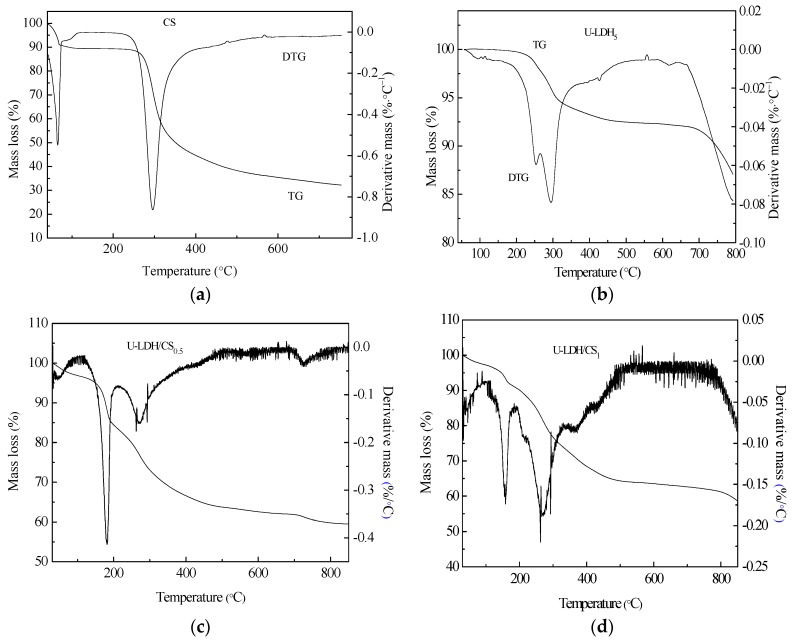

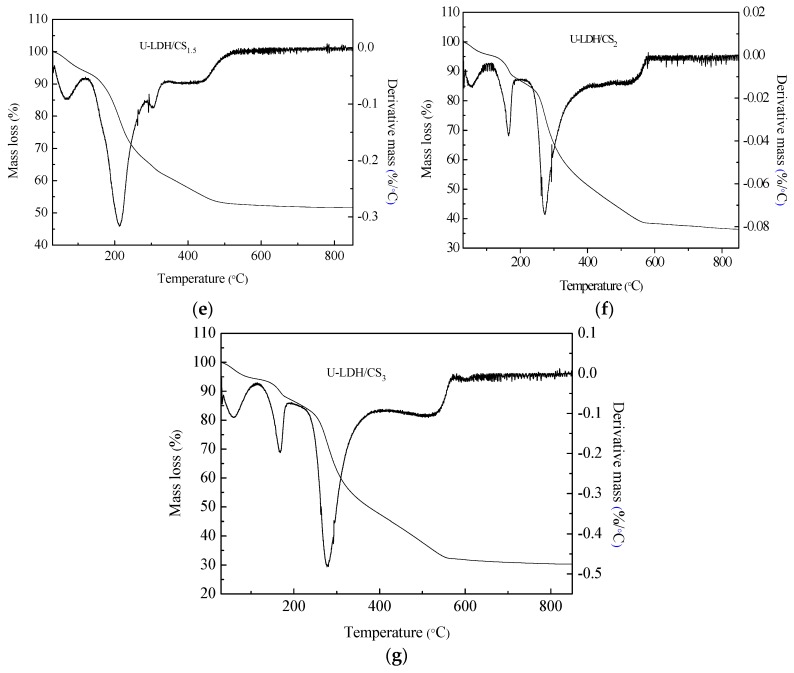
Thermal gravimetric and differential thermal gravimetric TGA-DTG curves of (**a**) pure CS; (**b**) U-LDH_5_; (**c**) U-LDH/CS_0.5_; (**d**) U-LDH/CS_1_; (**e**) U-LDH/CS_1.5_; (**f**) U-LDH/CS_2_; (**g**) U-LDH/CS_3_.

Thermogravimetric Analysis (TG/DTG) is a thermal analysis technique that analyzes the composition and structure of materials and their thermal stability by measuring the relationship between mass loss and temperature during temperature control. To understand the thermal stability of CS, U-LDH_5_, and U-LDH/CS with different CS concentrations, we performed TG/DTG analysis of each sample. Figure 8a–g shows the TG/DTG curves of the CS, U-LDH_5_, and U-LDH/CS samples. The hydration properties of CS polysacharides depend on primary and supra macromolecular structure [43]. The decomposition of chitosan is presented in two stages, the first one, which occurred at 50 °C and extended to about 75 °C, was due to loss of water molecules, with a weight loss of about 9.3% [44], followed by stability from 75 °C to about 250 °C. Then a second stage, corresponding to the primary degradation of the pure CS, happened at 250 °C, with a percentage weight loss of about 49.3% from 250 to 550 °C, which was similar to the literature [43]. A slow degradation trend occurred after550 °C that stabilized as it approaches 800 °C. Generally, in CS, the decomposition process of the N-acetylated compound is overlapped by the N-deacetylated unit, thereby increasing the widening process seen at temperatures up to 400°C [45], CS had a total mass loss of approximately 65.6%.

As can be seen from Figure 8b, the U-LDH_5_ thermal decomposition process is divided into three stages [46,47], between 247~300 °C for the first phase of weight loss, which is due to the removal of water from the interlayer and the physical adsorption surface [48]. At this step, U-LDH_5_ remained a layered structure. The second stage of weight loss, between 300 and 450 °C, was due to the decomposition of the interlayer ${\mathrm{CO}}_{3}^{2-}$ and the removal of the hydrotalcite-plate-OH, marking the delamination of the layered structure [49]. The last weight lost, that can be attributed to the decomposition of carbonate ions in the interlayer, started after 700 °C [50]. From Figure 8b, the mass loss of U-LDH_5_ was 6% in the first stage, corresponding to a maximum endothermic peak of 300 °C for DTG. The mass loss of the second phase of 1.5% corresponded to an observed edge of DTG at 448 °C. The last stage of U-LDH_5_ weight loss presented an edge at 570 °C, corresponding to a 8.2% weight loss with a total weight loss ofU-LDH_5_ of about 16.5%. The TGA-DTA curves of U-LDH/CS at different CS concentration, shown in Figure 8c–ghave a quite similar trend with a difference in weight loss and endothermic peaks range. U-LDH/CS_0.5_ showed a first mass loss of 2% at 75 °C between 50 and 102 °C, which can be accredited to the loss of adsorbed water, followed by a second stage of weight loss of U-LDH/CS_0.5_ from 150 °C to 450 °C of 30%, with the maximum endothermic peak at 180.6 °C. U-LDH/CS_0.5_ weight loss of the third stage was 4.07%, with the maximum endothermic peak at 650.1 °C, and the total weight loss was 36.7%. U-LDH/CS_0.5_ TGA showed a gradual stabilization after 700 °C, which proceeded a new slight weight loss of mass then a definitive stabilization up to 800 °C. The first and second phases of U-LDH/CS_1_ mass loss were 8.36% (40–190 °C) and 26.72% (200–450 °C), respectively, corresponding to the maximum endothermic peak at 163.4 and 275.9 °C. The third and last phase was 5.01% at 750 °C, corresponding to an extension of the primary degradation of the pure CS associated with decomposition of the hydrotalcite interlayer. The total weight loss of U-LDH/CS_1_ was 40.09%. It was also noted that the total mass losses of U-LDH/CS_1.5_, U-LDH/CS_2_, and U-LDH/CS_3_ were 46.3%, 51.3%, and 68.4%, respectively, distributed over two phases. These phases corresponded to a loss of water molecules, similar to the first phase of degradation of chitosan for the first stage, and the second stage was due to the primary degradation of the pure CS associated with decomposition of the interlayer and carbonate ions of hydrotalcite. The DTG curve of U-LDH/CS_1.5_ had two maximum endothermics peaks at 75 °C over the range 50–150 °C and the second at 220 °C between 150 and 250 °C in addition to two edges at 260 and 460 °C. The DTG curves of U-LDH/CS_2_ and U-LDH/CS_3_ exhibited three maximum endothermic peaks in the same temperature ranges and one edge each. Their maximum endothermic peaks were at the same locations at 75 °C, 180 °C, and 280 °C in the interval ranges of 50 to 100 °C, 100 to 200 °C, and 200 to 300 °C, respectively. It was only the edges whose positions were different; one at 550 °C for U-LDH/CS_2_ and the other at 530 °C for U-LDH/CS_3_. TG/DTG curves showed that CS molar ratio changes had a strong effect on the thermal stability of the hybrid, indicating that even though the CS molar ratio increased, the charge density of the laminate decreased, but the hydrogen bonding between the interlayer H_2_O and the interlayer ${\mathrm{CO}}_{3}^{2-}$, OH- anion, the interlayer -OH, and the interlayer anion interactions were not affected much. It is worth mentioning that U-LDH/CS_2_ and U-LDH/CS_3_ showed a strong phase of weight loss at 200–550 °C, with a mass loss of 48.6% and 51.3%, respectively. These losses may be due to the hybrid U-LDH/CS arrangement during the formation of spinel transformation given the high concentration of CS. Obviously, increasing the concentration of CS for the preparation of U-LDH/CS induced the decrease of the thermal resistance of the U-LDH/CS hybrid.

### 3.2. Antimicrobial Activity

**Figure 9:**
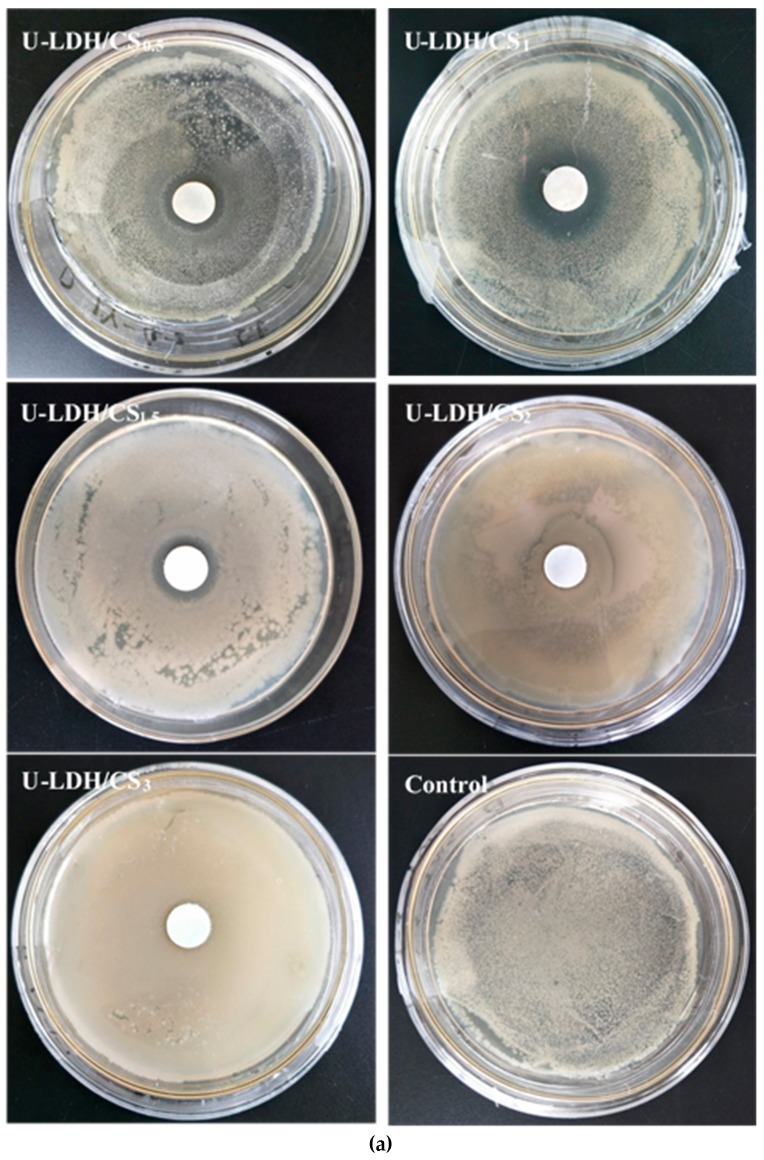

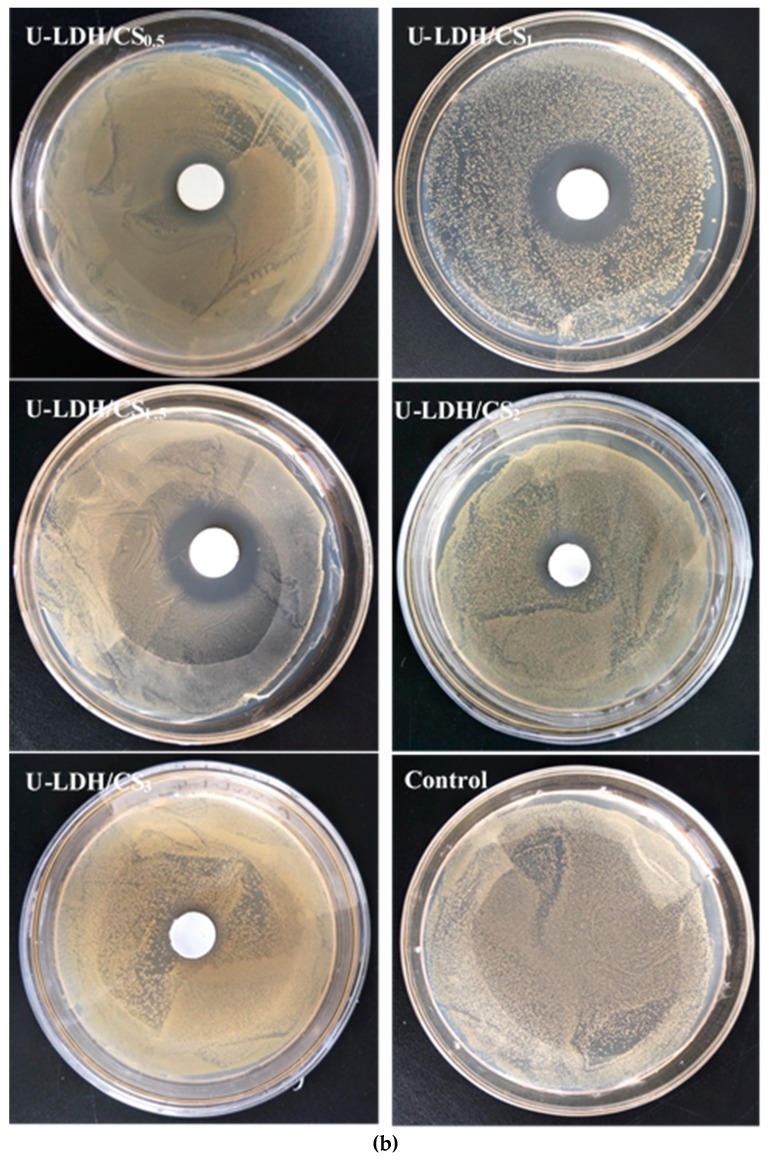

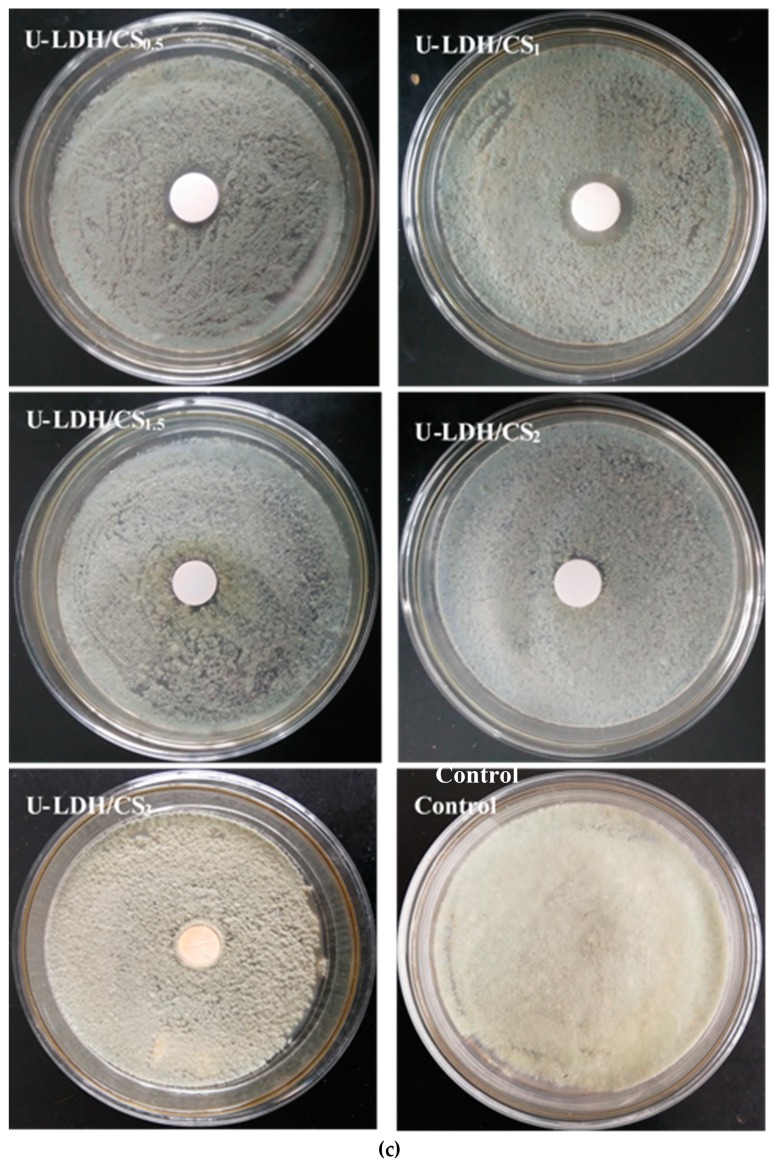
Inhibition zones of the U-LDH/CS samples and control against (**a**) *E. coli.*; (**b**) *S. aureus*; (**c**) *P. cyclopium*.

The antimicrobial activities of the pure CS and U-LDH/CS hybrids prepared in different CS concentration were measured according to the inhibition zone diameter method, and the results are shown in Figure 9a–c. All samples were tested against *E. coli*, *S. aureus*, and *P. cyclopium*. As seen in Figure 9a–c and Table 2, the control, pure CS (Figure S1), and U-LDH/CS_3_ meshes did not display antimicrobial activity, while the U-LDH/CS_0.5_ and U-LDH/CS_1_ meshes exhibited strong antimicrobial activity against the three microorganisms. In particular, U-LDH/CS_1_ showed the highest antimicrobial ability and produced average inhibition zones of 24.2, 30.4, and 22.3mm against *E. coli*, *S. aureus*, and *P. cyclopium*, respectively. The antimicrobial activity of U-LDH/CS_1_ was much larger than that of U-LDH_5_ due to the incorporation of CS molecules. The results suggest that the hybrids of chitosan and ZnAl-LDH made for the improvement of antimicrobial ability.

The antimicrobial inactivity of pure chitosan was reported by Kong et al. in this term: “The antimicrobial activity for chitosan is pH dependent”. The ability of chitosan to inhibit microbial growth is observed only in an acidic medium, where the polymer is soluble and carries a net positive charge [51,52]. The failure of chitosan to remain bactericidal at pH values of around six could be explained by the presence of a large majority of positively uncharged amino groups as well as by the poor solubility of chitosan [53,54]. The pH of the bacterial culture medium (pH 5.7~7.5) inhibits the chelating effect that could cause the chitosan on the bacteria to cause their lysis. Therefore, to overcome this limit, derivatization of chitosan is particularly aimed at enhancing the solubility of chitosan in aqueous medium while improving chitosan antimicrobial properties. As an example, Kong et al. [55] proposed the use of chitosan microsphere (CM) in a solid dispersing system, and even in this case, chitosan, which showed inhibitory effects, had a deacetylation degree DD of 62.6%. Above this value, and especially as reported by Kong et al. [55], in the range 83.5 to 97.5 of DD, CM showed no antimicrobial activity. Furthermore, chitosan with a lower degree of acetylation (DA) resulted in increased antimicrobial activity against various strains of fungi, Gram-positive, and Gram-negative bacteria [56]. The chitosan used in this study had a DA of 91%, which is very high. The lack of antimicrobial activity shown by chitosan could be made worse by the large size of pure chitosan particles, which prevents their entry through the pores of the bacterial membrane. Hence, the inability to cause membrane lysis as might be expected. This explains the importance of hydrolyzing chitosan in order to reduce its molecular and voluminous mass and also associate it with other compounds to protect it, thus enabling it to conserve its electrical charge. The significant antibacterial activity of the U-LDH/CS >> U-LDH >>> CS hybrid is due to the combination of the antibacterial activity of chitosan now protected by its association with the U-LDH and the antibacterial activity of the latter. Using protecting groups presents advantages, such as allowing selective modifications at the reactive centers, allowing reactions in the homogeneous medium, and giving a high degree of substitution in the products [57]. Indeed, the introduction of functional groups, such as trimethyl or quaternary alkyl groups, gives the polymer a permanent positive charge, improving its solubility in an aqueous medium and making it possible to measure the bioactivity at pH 7 [23].

In Figure 9a, against Gram-negative *E. coli*, the antibacterial activity is marked by the translucent outline around the material hybrid. This can be supported by the mechanism proposed by Dutta et al. [58]. The inactivation of *E. coli* by chitosan occurred via a two-step sequential mechanism; an initial separation of the cell wall from its cell membrane, followed by destruction of the cell membrane. This is confirmed by the mechanisms proposed by Zeng et al. and Papineau et al. [26,59]. Chitosan, through the hybrid, was reactivated and acted mainly on the outer surface of the bacteria. At a lower concentration (0.2 mg/mL), the polycationic chitosan does probably bind to the negatively charged bacterial surface to cause agglutination, while at higher concentrations the larger number of positive charges may have imparted a net positive charge to the bacterial surfaces to keep them in suspension. In Figure 9b, against Gram-positive *S. aureus*, the visible translucent areas are the inhibitions zones, which do not results in the behavior of the hybrid U-LDH/CS towards the bacteria strain. The hybrid U-LDH/CS on the surface of the cell (*S. aureus*) can form a polymer membrane, which inhibits nutrients from entering the cell [26]. The hybrid mechanism on fungi (*P. cyclopium*), shown in Figure 8c, can be explained by the mechanism described by Bai et al. [60]. Chitosan, after being protected by U-LDH, activated a dual function: (a) to direct the interference of fungal growth and (b) to activate several defense processes. The strong antimicrobial activity shown by the U-LDH/CS hybrid results from a synergistic harmony of the proportions of ZnAl-LDH and CS compounds, and the adequation of their combination has activated the activity of the antimicrobial compounds CS, Zn^2+^, ZnO, and Al_2_O_3_ contained in the structure of the hybrid. This could lead to the probable antimicrobial activity mechanisms of the hybrid U-LDH/CS described below.

**Figure 10:**
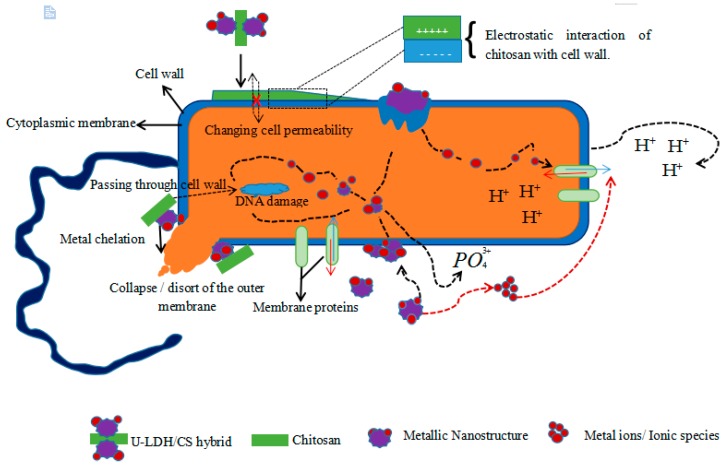
Schematicrepresentation of probable mechanisms involved in U-LDH/CS antimicrobial activity.

The antimicrobial mechanism of the hybrid could be compared to that of the chitosan derivative as described by Hosseinnejad and Jafari [61]. It is a multiple action posed by all components of the hybrid taken together or individually. The chitosan molecules of the hybrid, positively charged (cationic NH^3+^ groups), interfere with negatively charged bacterial cell membranes. This interaction with the bacteria membrane leads to the alteration of cell permeability and membrane lysis [62,63,64]. The hybrid, via chitosan, could activate a chelation of nutrients, causing the inhibition of microbial growth by the essential metals [63,65]. The hybrid could also, through the generation of reactive oxygen species (ROS) through its metallic compounds, cause lysis of the bacterium [66]. The production of ROS is presented as a major contributor to the antibacterial activities of various metal oxides [67]. Such reactive species are superoxide anion (O^2^), hydrogen peroxide (H_2_O_2_), and hydroxide (OH-). The toxicity of these species involves the destruction of cellular components such as lipids, DNA, and proteins as a result of their internalization into the bacteria cell membrane [61]. Beside these mechanisms, it also causes the release of zinc ions (Zn^2+^) in medium containing ZnO nanoparticles and bacteria [68,69]. Released Zn^2+^ has a significant effect, causing active transport inhibition as well as the disruption of the amino acid metabolism and enzyme systems [70,71]. The probable antimicrobial activity mechanisms of hybrid U-LDH/CS is proposed in Figure 10.

U-LDH/CS_2_ and U-LDH/CS_3_ showed either no antimicrobial effect, or a decreased antimicrobial effect. The characterization analysis (UV-Vis, thermogravimetric, and rheological) showed that U-LDH/CS_2_ and U-LDH/CS_3_ behaved almost like pure chitosan. It seems that at about 2 g L^−1^ or more of chitosan concentration, U-LDH_5_ is either insufficient or unable to firmly attach and protect the chitosan against the pH of the medium. LDH, which has not been attached to chitosan, has therefore not been able to release in the medium metal oxides and ionic particles. So, the chitosan molecules that are subjected to the pH of the medium diffuse this pH effect by their interconnection bond, which considerably reduces the chitosan molecules that have retained their antimicrobial activity. In addition, the large amount of chitosan also disrupts the antibacterial activity of metallic nanostructures released from LDH. This explains why, from 2 g L^−1^ of chitosan concentration, there is a remarkable reduction or loss of the antimicrobial activity of chitosan. This was confirmed by rheological, FTIR, UV-Vis, and thermogravimetric analyses, which indicated that U-LDH/CS_3_ showed fragility to remain compact, with U-LDH/CS_2_ and U-LDH/CS_3_ absorption bands being more similar to those of chitosan. The amount of chitosan is a significant factor of effectiveness of the antibacterial activity of the hybrid. In the range of 0.5 to 1.5 g L^−1^ of chitosan concentration, its plays a stimulating role in the antimicrobial activity of the hybrid by producing an increase of the antimicrobial activity. This increase of the hybrid antimicrobial activity is shown by the enlargement of the inhibition zones. However, in the range of 1.5 to 3 g L^−1^, chitosan concentration has an inhibitory effect on the antimicrobial activity of the hybrid. This results in a reduction or complete loss of the antimicrobial activity of the hybrid. The concentration of chitosan therefore has a pivotal role in the synthesis and efficacy of the antimicrobial activity of the U-LDH/CS hybrid.

## 4. Conclusions

The development and spread of antibiotic-resistant pathogens involves research to develop new antimicrobial agents. Hybrids derived from chitosan are newly emerging areas of research into the synthesis of antimicrobial agents. Hybridization of chitosan with ZnAl-LDH has the potential to restore the effectiveness of the antibacterial activity of chitosan at a pH of about six and above, provide novel candidates with a synergistic effect in terms of efficacy and lowered resistance selection propensity, and could confer antibacterial activity against a broad spectrum of resistant microbes. Antibacterial hybrid materials based on the ZnAl/chitosan matrix have been successfully synthesized. It was shown that the obtained hybrid tested against *E. coli*, *S. aureus*, and *P. cyclopium* demonstrated the highest antimicrobial activity toward *S. aureus*. It has been found that an increase of CS concentration around and above 1.5 g L^−1^ led to a decrease in the antibacterial activity of U-LDH/CS. The effectiveness of the antibacterial activity of U-LDH/CS hybrids suggest that the proposed preparation of such hybrid materials could be extended to the development of other organic-inorganic hybrid antimicrobial materials that may have applications in several areas of biotechnology and medical engineering.

## Figures and Tables

**Table 1:** Calculation of lattice parameters and basal plane for the U-LDH_5_, U-LDH/CS_0.5_, U-LDH/CS_1,_ U-LDH/CS_1.5_, U-LDH/CS_2_, U-LDH/CS_3_.

Samples Reflections	U-LDH_5_	U-LDH/CS_0.5_	U-LDH/CS_1_	U-LDH/CS_1__.5_	U-LDH/CS_2_	U-LDH/CS_3_
*d*_003_ (nm)	0.7691	0.7627	0.7680	0.7596	0.7659	0.7568
*d*_006_ (nm)	0.3828	0.3803	0.3818	0.3795	0.3813	0.3785
*d*_009_ (nm)	0.2487	0.2595	0.2597	0.2594	0.2598	0.2586
*d*_110_ (nm)	0.1631	0.1635	0.1630	0.1635	0.1636	0.1630
FW_003_ (rad)	0.4080	0.2240	0.2560	0.2990	0.260	0.5100
FW_110_ (rad)	0.7770	0.4760	0.1810	0.6500	0.79	0.6700
*a* (nm)	0.3262	0.3204	0.3895	0.2493	0.1640	0.9613
*c* (nm)	2.3073	2.2471	2.2743	2.2802	2.2097	2.2174

**Table 2:** Antimicrobial activities of the U-LDH/CS hybrids.

Samples g	*E. coli*	*S. aureus*	*P. cyclopium*
**Control**	0 mm	0 mm	0 mm
**Pure CS**	0 mm	0 mm	0 mm
**U-LDH_5_**	16.5 ± 0.3 mm	20.5 ± 0.6 mm	9.8 ± 0.7 mm
**U-LDH/CS_0.5_**	18.3 ± 0.4 mm	23.2 ± 0.2 mm	13.3 ± 0.7 mm
**U-LDH/CS_1_**	24.2 ± 0.8 mm	30.4 ± 0.5 mm	22.3 ± 0.5 mm
**U-LDH/CS_1.5_**	17.1 ± 0.3 mm	27.0 ± 0.6 mm	0mm
**U-LDH/CS_2_**	0 mm	14mm	0 mm
**U-LDH/CS_3_**	0 mm	0 mm	0 mm
Tetracycline*	21 ± 0.9mm	26 ± 0.9 mm	21 ± 0.8 mm

Notes: Values are mean of three replicates; * standard antibiotic.

## Supplementary Materials

The following are available online at https://www.mdpi.com/2073-4360/11/10/1588/s1.
