# Supplementary figures and images for: Preparation of the Hybrids of Hydrotalcites and Chitosan by Urea Method and Their Antimicrobial Activities
Source: Polymers (Basel). 2019 Sep 28;11(10):1588. doi: 10.3390/polym11101588 (PMC6835444; doi:10.3390/polym11101588)

## Supplementary material

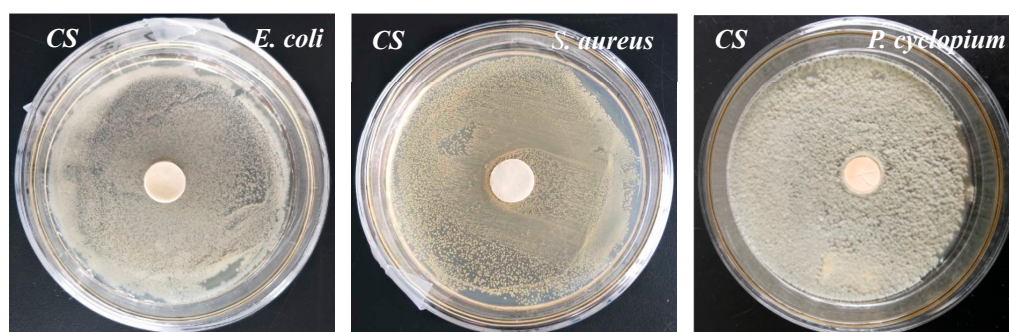

**Fig s1** Inhibition zones of the pure chitosan

Supplement: Supplementary file 1 [file polymers-11-01588-s001.pdf]
